# Evaluation of the association between exosomal levels and female reproductive system and fertility outcome during aging: a systematic review protocol

**DOI:** 10.1186/s13643-019-1228-9

**Published:** 2019-11-28

**Authors:** Halimeh Mobarak, Reza Rahbarghazi, Francesca Lolicato, Mohammad Heidarpour, Fariba Pashazadeh, Mohammad Nouri, Mahdi Mahdipour

**Affiliations:** 1 Department of Clinical Sciences, Faculty of Veterinary Medicine, Ferdowsi University of Mashhad, Mashhad, Iran; 2 Stem Cell Research Center, Tabriz University of Medical Sciences, Tabriz, Iran; 3 Department of Applied Cell Sciences, Faculty of Advanced Medical Sciences, Tabriz University of Medical Sciences, Tabriz, Iran; 4 Follicle Biology Laboratory, UZ Brussel, Vrije Universiteit Brussel, Brussels, Belgium; 5 Research Center for Evidence-Based Medicine, Health Management and Safety Promotion Research Institute, Tabriz University of Medical Sciences, Tabriz, Iran; 6 Department of Reproductive Biology, Faculty of Advanced Medical Sciences, Tabriz University of Medical Sciences, Tabriz, 5166615739 Iran

**Keywords:** Extracellular vesicles, Exosomes, miRNAs, Female reproduction, Fertility, Aging, Systematic review

## Abstract

**Background:**

Exosomes may have critical roles in the maternal-embryo cross-talk for the recognition and maintenance of pregnancy during maternal aging. Exosomes have the capability to carry developmental signaling molecules with the ability to modulate gene expressions and affect growth and regulation of embryo during pregnancy. Systematic review aims to evaluate age-related alterations in the exosomal content and functions that can influence the reproductive performance in human and animal models as conveyors of senescence signals.

**Methods:**

A literature search of electronic databases including MEDLINE (PubMed), Embase, ProQuest, Scopus, Google Scholar, WHO, SID, MAGIRAN, and Barakat will be conducted. Following the online search, articles will be screened by two independent reviewers according to inclusion and exclusion criteria. Eligible studies will be critically appraised by reviewers at the study level for methodological quality using Joanna Briggs Institute’s standardized critical appraisal tools. The extracted data from selected studies will cover the study populations, methods, current evidence about the physiological role of extracellular vesicles and exosomes in reproductive system, relevant outcomes, and possible conclusions about the effectiveness of exposure.

**Discussion:**

Regarding the role of exosomes and their cargoes in the function of reproductive tract, the possible beneficial or adverse effects following exosomal administration from younger women to older women will be evaluated in the systematic review. As a result, exosome therapy could be suggested as a novel therapeutic agent if the favorable results are identified.

## Background

Interplay between cells is a critical factor in coordinating cellular events in all living organisms and accomplished partially by engaging different mechanisms mediated by the release and uptake of extracellular vesicles (EVs) [1–4]. EVs, a heterogeneous population of nanoscale vesicles, are secreted by cells in an evolutionarily conserved manner in all the organisms [3, 5]. These vesicles are capable of transmitting genetic information and delivering molecular signals to the recipient cells, affecting their functions by either induction of surface ligands or transferring molecules implicated in diverse physiological and pathological processes [3, 6–8]. A well-recognized systematic classification divided the EVs into exosomes and microvesicles (MVs) as well as apoptotic bodies (Abs), based on their biogenesis, size, and content [9–11].

Due to diagnostic and therapeutic potential in different diseases, exosomes have raised increasing attention among researchers over the last two decades [12]. Exosomes are specialized lipid bilayer nanovesicles released by a variety of cell types with capacity in transferring biologically active macromolecules, such as RNAs (including coding and non-coding RNAs), proteins, and lipids [7, 10, 13].

Exosomes are generally recognized as potent paracrine mediators of cellular communication and identified in numerous biological fluids. Various functions in cellular maintenance, translational activity, cell proliferation, and apoptosis, tissue repair, angiogenesis, metabolism, antigen presenting, immune modulation in inflammation, reproduction, blood coagulation, tumor pathogenesis, and spread of inflammatory, autoimmune, neurodegenerative and infectious diseases have been addressed for exosomes [13–19].

The controlled biogenesis of exosome is a physiological phenomenon while the increased levels of exosomes have been correlated with various diseases and pathological conditions. Based on scientific literature, the healthy aging process can change the entity of circulating EVs, particularly the exosome biogenesis and even trafficking rate. Most of the studies demonstrated that exosomal changes have been associated with the aging process and the emergence of age-related diseases (ARDs) [16, 20–25].

The onset of reproductive decline correlates with genetic factors, lifestyle, age, and environmental changes [26, 27]. According to previously published data, reduced fertilization and pregnancy rates coincided with miscarriage possibility are shown by aging [17, 27–32].

Nowadays, women are aiming to get pregnant at high ages for a variety of reasons [33]. Researchers are looking for alternative procedures to improve fertility rate and raise the pregnancy chance in older women via assisted reproductive treatments and advanced therapeutic materials. Fertility and reproductive functions have an inevitable correlation with correct folliculogenesis, oogenesis, implantation, embryo development, and successful pregnancy which are influenced by cell-to-cell communication through extracellular vesicles (e.g., MVs, exosomes), proper cross-talk between mother and embryo during pregnancy as well as gene and protein expression levels in cells from reproductive system [15, 34, 35].

Numerous studies noted that exosomes are released from various parts of the female reproductive tract, including endometrium, oviducts epithelium, and uterine as well as preimplantation embryos, trophoblastic cells, and the placenta [18, 35–37]. It has been demonstrated that exosomes play a significant role in transporting molecular cargos having the potential to modulate transcription and translational activity, normal follicular growth, granulosa cells proliferation and differentiation, gametogenesis, oocyte maturation, fertilization rate, embryo development, blastocyst formation, implantation, and pregnancy outcome [1, 15, 38, 39].

Recent studies have compared exosomal miRNA profiles in ovarian follicular fluid, placenta and uterine discharge, and plasma isolated from young and old equine, bovine, and human samples. Following bioinformatic and experimental analysis, researchers revealed that these small non-coding RNAs could target effectors in signaling pathways such as mitogen-activated protein kinase (MAPK), transforming growth factor beta (TGF-β), wingless signaling pathway (Wnt), neurotrophin, insulin signaling pathways, epidermal growth factor receptor (ErbB) pathways, and factors related to ubiquitin-mediated pathways inside exosomes. These factors correlate with follicular growth, meiotic resumption, oocyte maturation, and ovulation [34, 40–43]. In addition, exosomal miRNAs could be considered as potential and novel biomarkers for age-related decline of oocyte competence and quality [34, 42]. The identification of exosomes in reproductive system secretome highlights their possible roles in intercellular communications required for pre- and post-conception during advanced maternal aging. Alterations in the quality and quantity of exosomes in humans, however, have recently been used as a hallmark for pregnancy and pregnancy-related pathologies [44, 45].

Herein, we decided to systematically review the current data on the EVs with particular attention to exosomes under the physiological and biological conditions as well as signaling pathways associated with aging in cells and organism which could affect the female reproductive system and pregnancy outcome during maternal aging.

## Methods/design

The systematic review manuscript will be prepared according to the standards of the Preferred Reporting Items of Systematic Reviews and Meta-Analysis Protocols (PRISMA-P) statement and checklist, and Preferred Reporting Items for Systematic Reviews (PRISMA) guidelines [46, 47]. See Additional file 1: PRISMA-P 2015 Checklist.

### Inclusion criteria

#### Studies designs

##### Type of studies

This review will consider observational reports including prospective, retrospective cohort, case-control, and analytical cross-sectional studies and will also consider descriptive observational studies including case series, individual case reports, and descriptive cross-sectional analysis for inclusion. All relevant articles which have examined the role of exosomes in every aspect of female reproductive system and report exosomal level alterations or their contents during the aging in reproductive-related biofluids will be included in the systematic review.

##### Participants

Female sexes in both human and animal studies will be considered.

##### Exposure/assessment

This review will consider studies that evaluate exosomal levels or contents such as miRNAs and mRNAs in reproductive biofluids evaluated in young and old age groups.

##### Outcomes

During the systematic review, the following outcomes of fertility rate, implantation rate, oocyte quality improvement and maturation rate, growth, and maturity of germ cells and early embryonic development, will be evaluated. In order to investigate the possible association between fertility and reproductive outcome with exosomal dynamics, the analyzed exosomal content and levels in young and old group of subjects will be correlated to their respective fertility outcomes.

### Search strategy

The search strategy will aim to recognize both groups of published and unpublished studies. An initial limited search of Embase and MEDLINE (via PubMed ) was carried out to identify relevant keywords including index terms and free text words as a starting point for construction and validation (Appendix: Initial search strategy for Embase). Then, these keywords will be used during final search process in bibliographic databases that will be adapted for each information sources accordingly. To avoid any missing documents, the reference list of all studies will be appraised. Articles published in English will be included in the systematic review. Studies presented in different languages other than English will be excluded.

### Information sources

Electronic databases include MEDLINE (PubMed), Embase, and Scopus. Unpublished studies within the gray literature category, including ProQuest for dissertation and thesis, Google Scholar, WHO (World Health Organization), SID, MAGIRAN, Barakat will be as well-considered.

### Study selection process

Following the search, all identified citations will be exported into EndNote X7.2.1 and duplicate studies will be removed. Titles and abstracts will then be screened by two independent reviewers for assessment against the inclusion criteria for the review (all relevant articles that examined the role of exosomes in all aspect of reproductive system and also exosomal levels or their contents during the aging process in the female human or animal groups). Potentially relevant studies will be retrieved in full-text and their bibliography details will be monitored. The full text of selected documents will be assessed in detail against the inclusion criteria by two independent reviewers. Reasons for exclusion of full-text studies that do not meet the inclusion criteria will be recorded and reported in the systematic review. See Additional file 2: PRISMA Flow Diagram. Any disagreements that arise between the reviewers at each stage of the study selection process will be resolved through consensus, or in consultation with a third reviewer.

### Data collection process

Data will be extracted from included studies by two independent reviewers using the standardized and modified Joanna Briggs Institute (JBI) data extraction tools [48]. The extracted data will include specific relevant information about the study populations, study methods, current evidences about the physiological role of extracellular vesicles and exosomes in reproductive system and outcomes related to the review’s specific objectives as well as authors conclusions about the effectiveness of exposure where possible. The favorable outcomes will cover fertility rate, implantation rate, oocyte improvement and maturation rate, growth, and maturity of germ cells and early embryonic development. Two independent reviewers will participate in the process of reading and selecting the data and the third reviewer in case of any disagreements. Authors of the nominated papers will be contacted to request for any missing or additional data, where required.

### Quality and risk of bias assessment

Eligible studies will be critically appraised by two independent reviewers at the study level for methodological quality using standardized critical appraisal instruments from the Joanna Briggs Institute for observational studies [49, 50]. Any disagreements that arise between the reviewers will be resolved through discussion, or in consultation with a third reviewer. The results of critical appraisal will be reported in a narrative form and in a tabular format. All studies, regardless of the results of their methodological quality, will undergo data extraction and synthesis (where possible).

### Data synthesis

To synthesize the extracted data, we will have two approaches for both qualitative and quantitative outcomes. For non-quantitative outcomes (e.g., oocyte quality improvement, growth, maturity of germ cells, and early embryonic development), the results will be reported descriptively (narratively in tabular and figure formats in data presentation). In case of quantitative outcomes (e.g., fertility, implantation, and oocyte maturation rates), if the included quantitative data provide sufficient material to synthesis and contain the required homogeneity, will be analyzed using CMA 2.1 software. The effect sizes will be presented as dichotomous data (odds ratios or relative risk) and their 95% confidence intervals will be calculated for quantitative data analysis. Heterogeneity will be assessed statistically using the standard chi-squared and *I*^2^ tests. Statistical analyses will be performed using random or fixed effects based on results [48, 51]. Additionally, studies will be pooled for human and animal studies, individually prior to the analysis.

A funnel plot will be generated using Comprehensive Meta-Analysis (CMA) software 2.1 to assess publication bias if there are 10 or more studies included in a meta-analysis. Statistical tests for funnel plot asymmetry (Egger test or Begg test) will be performed where appropriate.

## Discussion

To the best of our knowledge, no systematic reviews have been conducted on evaluation of the relationship between exosomal levels and female fertility outcome during aging. Identification of endogenous EVs such as exosomes in genital fluids could potentially illustrate their possible roles in the modulation of reproduction, growth, and maturation of germ cell and development of early embryo. These nano-sized microvesicles could be considered as a putative candidate biomarkers for pregnancy and pregnancy-related deficiencies. It will be anticipated that any changes in the amount and content of EVs such as exosomes could indicate the physiological or pathological changes in the genital organs (e.g., during aging) and may affect the performance of the reproductive system and eventually fertility outcome.

In the field of reproduction, the prospective systematic review will expand our knowledge on the possible role of exosomes in female reproductive system during aging changes. As the exosomes have prominent roles in various aspects of physiological activities including reproduction, one could hypothesize that these small vesicles might be of great interest for the researchers in the field of reproduction exploring the exosomal profile and contents within the genital tract (including miRNAs and other genetic factors, cytokines, growth factors, proteins).

## Supplementary information

**Additional file 1.** PRISMA-P 2015 Checklist

**Additional file 2.** PRISMA Flow Diagram; Study Selection Process Diagram

## Data Availability

Not applicable

## Appendix

**Table 1:** Example search strategy for Embase

Search	Query
#1	‘exosome’/exp
#2	‘exosome’:ab,ti
#3	#1 OR #2
#4	‘fertility’/exp
#5	‘fertility’:ab,ti
#6	‘nidation’/exp
#7	‘nidation’:ab,ti
#8	‘embryo implantation’:ab,ti
#9	‘oocyte’/exp
#10	‘oocyte’:ti,ab
#11	‘fetus maturity’/exp
#12	‘fetus maturity’:ti,ab
#13	‘germ cell’/exp
#14	‘germ cell’:ti,ab
#15	‘maturation’/exp
#16	‘maturation’:ti,ab
#17	‘growth’/exp
#18	‘growth’:ab,ti
#19	#15 OR #16 OR #17 OR #18
#20	#13 OR #14
#21	#19 AND #20
#22	#4 OR #5 OR #6 OR #7 OR #8 OR #9 OR #10 OR #11 OR #12 OR #21
#23	#3 AND #22
#24	‘aging’/exp
#25	aging:ab,ti
#26	#24 OR #25
#27	#23 AND #26

## Abbreviations

Abs: Apoptotic Bodies
ARDs: Age-related diseases
CMA: Comprehensive meta-analysis
ErbB: Epidermal growth factor receptor
EVs: Extracellular vesicles
MAPK: Mitogen-Activated Protein Kinase
MVs: Microvesicles
PRISMA: Preferred Reporting Items for Systematic Reviews and MetaAnalyses
PRISMA-P: Preferred Reporting Items for Systematic Review and Meta-Analysis-Protocols
TGF-β: Transforming Growth Factor beta
WHO: World Health Organization
Wnt: Wingless signaling pathway
